# Supplementary material for: Probabilistic modelling of effects of antibiotics and calendar time on transmission of healthcare-associated infection
Source: Sci Rep. 2021 Nov 1;11:21417. doi: 10.1038/s41598-021-00748-y (PMC8560804; doi:10.1038/s41598-021-00748-y)
Supplement: Supplementary file 1 — Supplementary Information. [file 41598_2021_748_MOESM1_ESM.pdf]

Probabilistic modelling of effects of antibiotics and calendar time on transmission of healthcare-associated infection

Mirjam Laager*[1], Ben S Cooper[1], David W Eyre[2] on behalf of the CDC Modeling Infectious Diseases in Healthcare Program (MInD-Healthcare)

[1]Nuffield Department of Medicine, University of Oxford, UK
[2]Big Data Institute, Nuffield Department of Population Health, University of Oxford, UK

*Corresponding author: mirjam.laager@unibas.ch

Supplementary Text: Accounting for readmissions

The model described in the main text assumes that patients do not loose colonisation while in the ward and that their probability of being positive on readmission is equal to the community prevalence and independent of the status on discharge of previous ward stays. These assumptions seem reasonable if the length of stay of patients is relatively short compared to the timescale at which loss of colonisation is expected and if not many patients are readmitted. Here we describe a version of the model where we account for loss of colonisation in the ward and explicitly model the probability of being positive on readmission by using the status on discharge of the previous ward stay and the time spent in the community. Assuming that loss of colonisation happens at a constant rate, the transmission likelihood can be modelled as

$$\Pi(S|\theta) = \left(\frac{p_c}{p_c + q_c}\right)^{N_p} \left(\frac{q_c}{p_c + q_c}\right)^{N - N_p}$$

$$\prod_{k=1}^{N}\left[\prod_{i=1}^{N_{a,k}} P_{ik}\right]\prod_{k=1}^{N}\left[\prod_{i=1,\, a_k^{i+1} - d_k^i > 1}^{N_{a,k}-1} M_{m_i n_k}^{a_k^{i+1} - d_k^i - 1}\right]$$

where S is a matrix containing the status of each patient during each day in the hospital, θ is a vector containing the transmission parameters, $N_{a,k}$ denotes the number of admissions of patient k and a_k^i and d_k^i are the first and last day of admission number i of patient k. The first two terms of the likelihood function capture the status of the patients before their first admission. The factors P_{ik} capture the daily probability of acquisition or loss of colonization while a patient is in the hospital. For patients who are readmitted the time between admissions is modelled using the transition matrix M which is defined below.
The status of a patient on the day before the first admission can be derived from the equilibrium prevalence in the community. For a constant community acquisition rate p_c and a constant community loss rate q_c the rate of change of the proportion of colonised individuals in the community is given by

$$\frac{dc(t)}{dt} = -q_c c(t) + p_c\big(1 - c(t)\big)$$

Neglecting the influx of colonised patients from the hospital into the community, we can assume that the probability of a patient being colonised on the day before admission is equal to the endemic equilibrium in the community.

The status of patients during their time in the ward is a product over all ward stays of all patients. The likelihood contribution of ward stay i of patient k is given by

$$P_{ik} = \prod_{j = a_k^i}^{d_k^i} \Big[(1 - q_w)^{1_{s_{k,j-1}=1, s_{k,j}=1}} \, q_w^{1_{s_{k,j-1}=1, s_{k,j}=0}} \, p_{kj}^{1_{s_{k,j-1}=0, s_{k,j}=1}} \, (1 - p_{kj})^{1_{s_{k,j-1}=0, s_{k,j}=0}} \Big]$$

where q_w denotes the constant loss rate in the ward and the entry s_{kj} of the matrix denotes the status of patient k on day j, which is 0 if the patient is susceptible and 1 if the patient is colonised. The first term of the product describes the probability of patient k remaining colonised on day j given that they are colonised on day $j - 1$. This is one minus the daily probability of losing colonisation in one day. The second term is for transitions from colonised to susceptible, which is given by the constant loss rate. The third and fourth term describe acquisition or no acquisition, with the daily probability of acquisition depending on the number of colonised patients in the ward and the antibiotics use as described above.
For patients who are readmitted the likelihood of changing or maintaining the status of the last day of the previous admission can be deduced using the one step transition matrix *M*, which is given by

$$M = \begin{bmatrix} 1 - p_c & p_c \\ q_c & 1 - q_c \end{bmatrix}$$

Raising the matrix *M* to the power of the number of days a patient spends in the community, which is given by $n = a_k^{i+1} - d_k^i - 1$, yields the likelihood of a patient being colonised or susceptible on readmission. Given the status on the last day of the previous admission and the status on readmission, the likelihood is the entry (m_i, n_k) of M^n where m_i and n_k are given by

$$m_i = \begin{cases} 1, if \; s_{k,d_k^i} = 0 \\ 2, if \; s_{k,d_k^i} = 1 \end{cases}$$

and

$$n_i = \begin{cases} 1, if \; s_{k,a_k^{i+1}} = 0 \\ 2, if \; s_{k,a_k^{i+1}} = 1 \end{cases}$$

If patients are readmitted to the hospital on the day after a discharge, we assume that they do not spend any time in the community, which is why the factor of the likelihood function which models acquisition and loss in the community is equal to one for patients where n is equal to zero.
We compared the model described in the main text to the model which accounts for readmissions by generating two datasets (data 1 and data 2) simulated under the same assumptions as in the model which does not account for readmissions (model 1) and the model accounting for readmissions (model 2). For the dataset with readmissions we sampled the number of admissions per patient from the empirical distribution of the readmissions in the

ICU dataset. The transmission parameters for both models were chosen such that the number of colonised patients were similar in both datasets. We inferred the model parameters of both models for both datasets and conducted 1000 forward simulations sampling from the posterior distributions. The results are shown in Supplementary Figure 1. The number of positive tests in the datasets lies within the interquartile range of the forward simulations for both models. From this we conclude that analysing the ICU dataset described in the main text of the paper by treating readmissions as new patients does not yield substantially different results with respect to the metrics used for the posterior predictive checks in the main text than when accounting for readmissions.

Supplementary Figures:

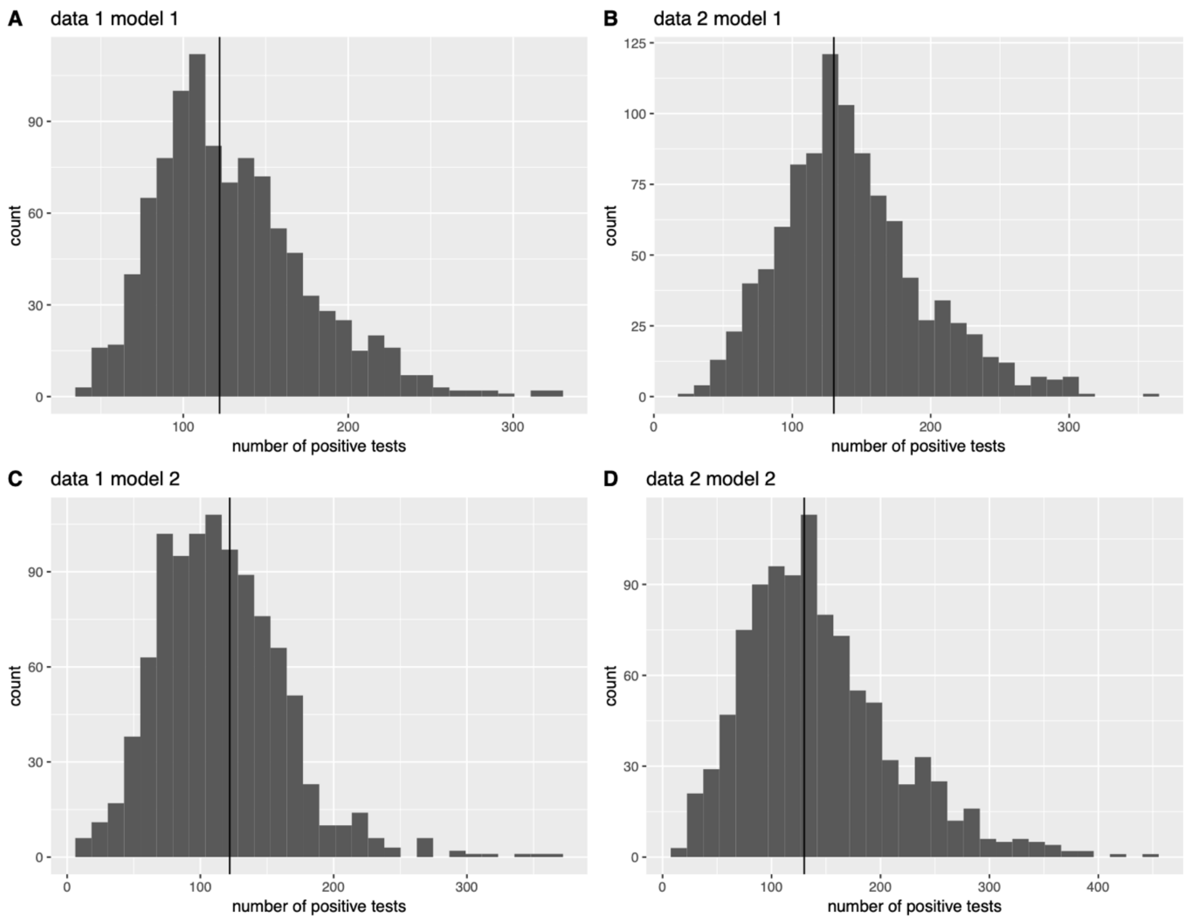

Supplementary Figure 1. Comparison of the model used in the main analysis with a model explicitly accounting for decolonisation and readmission. Simulated data was generated under the assumption that readmissions can be treated as new patients (data 1) or explicitly accounting for readmissions (data 2). Inference was conducted with the model used in the main analysis (model 1) and a model explicitly accounting for readmissions (model 2). The number of positive tests in the two datasets (solid vertical lines) is compared to the number of positive tests in 1000 simulations with parameter values drawn from the posterior distributions from model 1 (A and B) and model 2 (C and D).

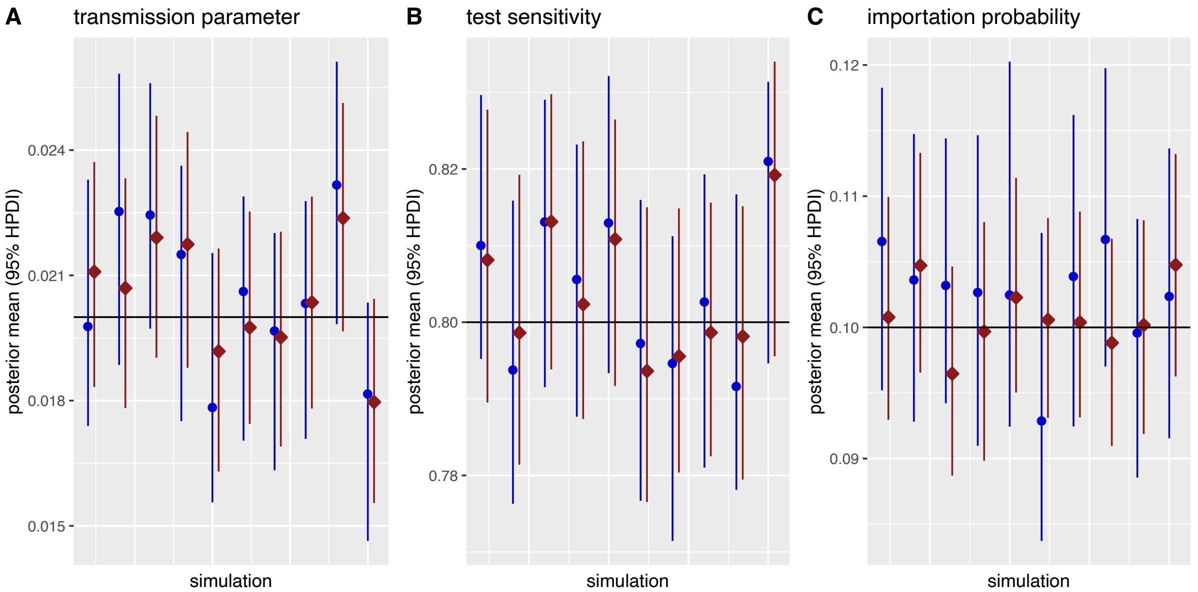

Supplementary Figure 2. Posterior estimates of the main model parameters in 10 simulated datasets. The true values are indicated by the solid horizontal lines. Each dataset was analysed with the model using positive and negative swabs only (blue circles) and using typing information (red squares). HPDI, highest posterior density interval.

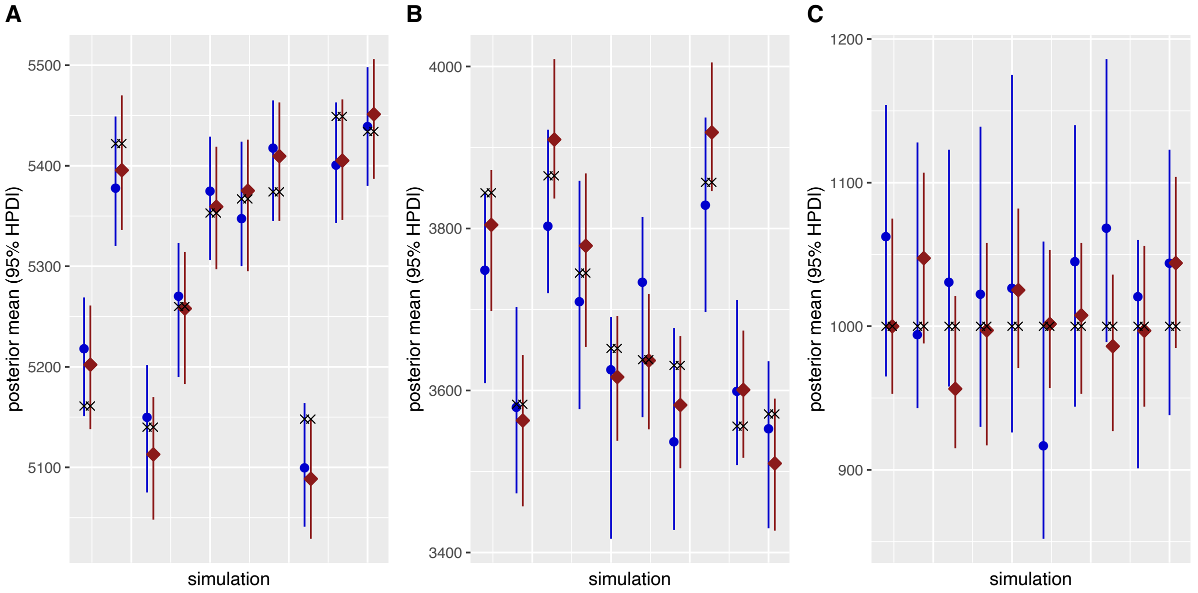

Supplementary Figure 3. Aggregated posterior estimates of the patient statuses in 10 simulated datasets. The true values of the total number of patients remaining susceptible during the entire admission (A), acquiring during the admission (B) and admitted already colonised (C) are indicated by the black crosses. Each dataset was analysed with the model using positive and negative swabs only (blue circles) and using typing information (red squares). HPDI, highest posterior density interval.

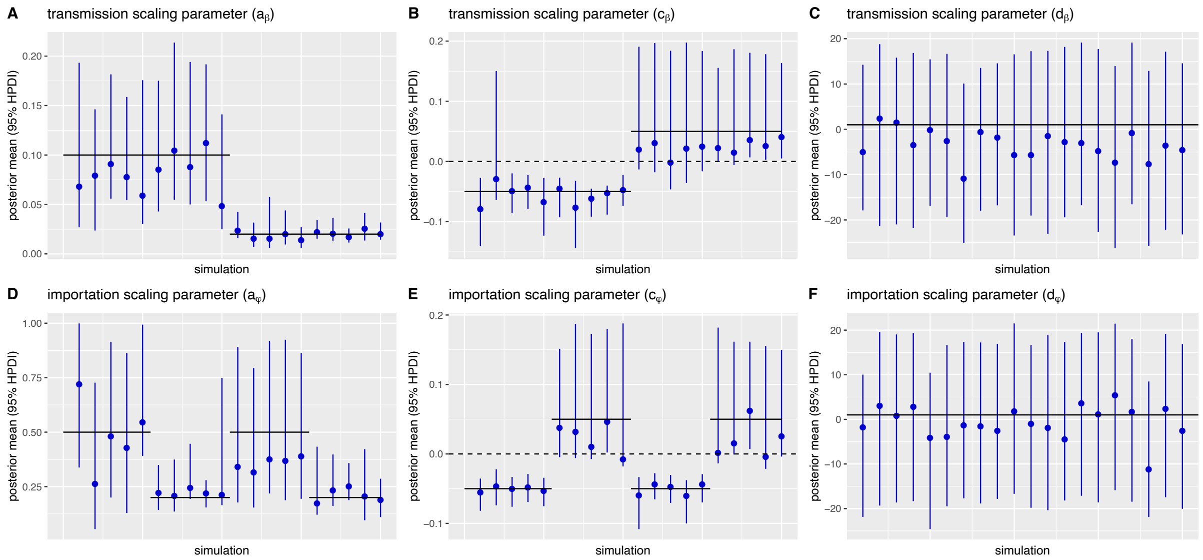

Supplementary Figure 4. Scaling parameters of time dependent transmission (A - C) and importation (D - E). Negative values of the slope parameter c (panels B and E) represent a decrease in transmission and importation respectively. Positive values represent an increase. The true values are indicated by the horizontal lines.

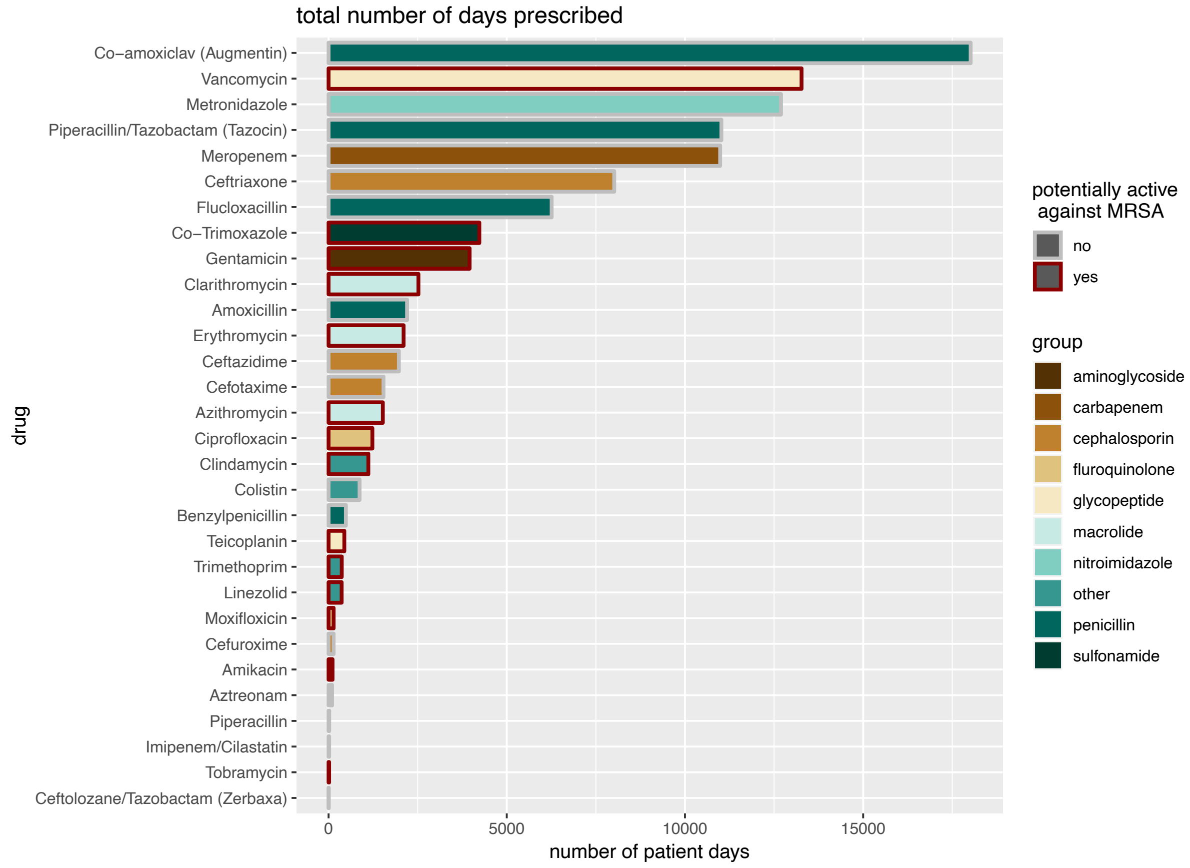

Supplementary Figure 5. Antibiotic exposures in 7924 ICU admissions. The total number of patient days in the dataset was 45331.

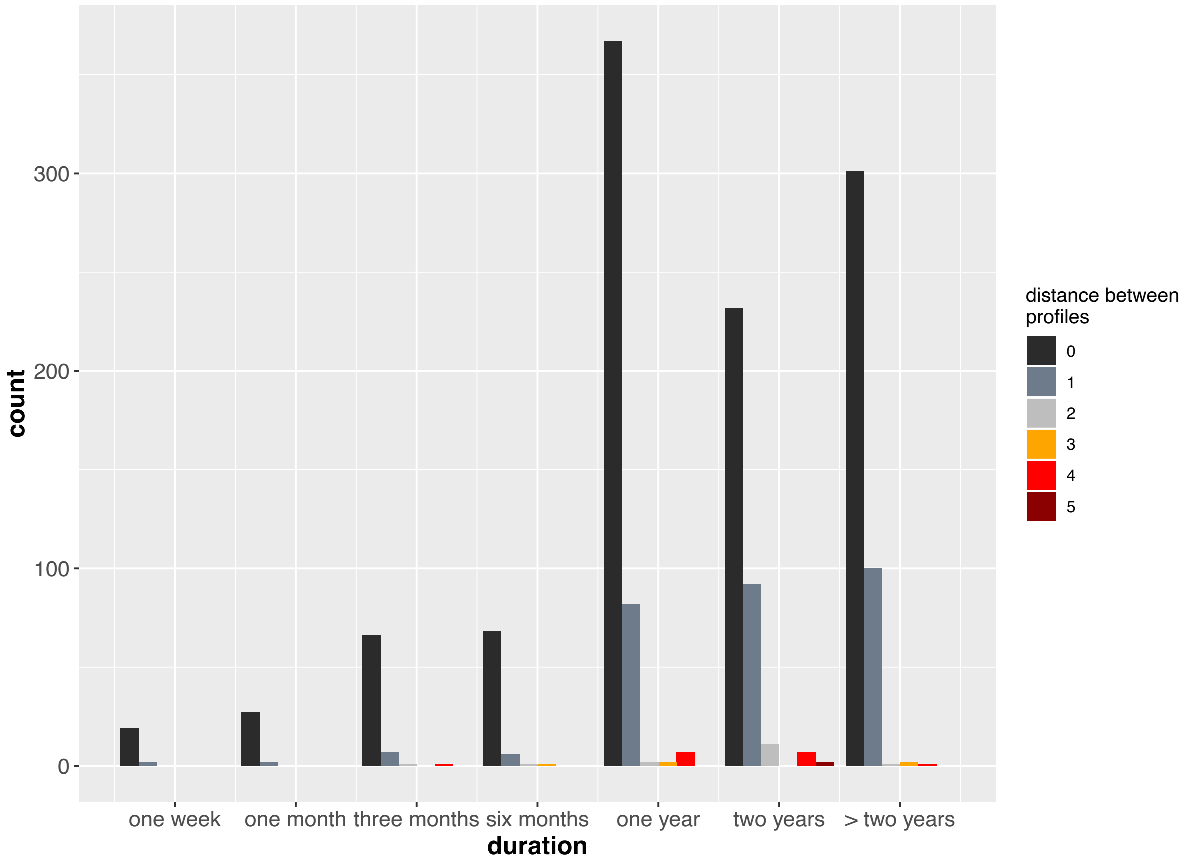

Supplementary Figure 6. Changes in resistance profiles over time. The resistance profiles were derived from the antibiotic resistance testing data based on 7 antibiotics (Gentamycin, Erythromycin, Tetracycline, Fusidic acid, Ciprofloxacin, Rifampicin, Mupirocin.). The duration between any two swabs from the same patient was grouped by the resistance profile distance between the two swabs. Distance is calculated by adding up the number of antibiotics for which the two profiles did not have the same resistance result.

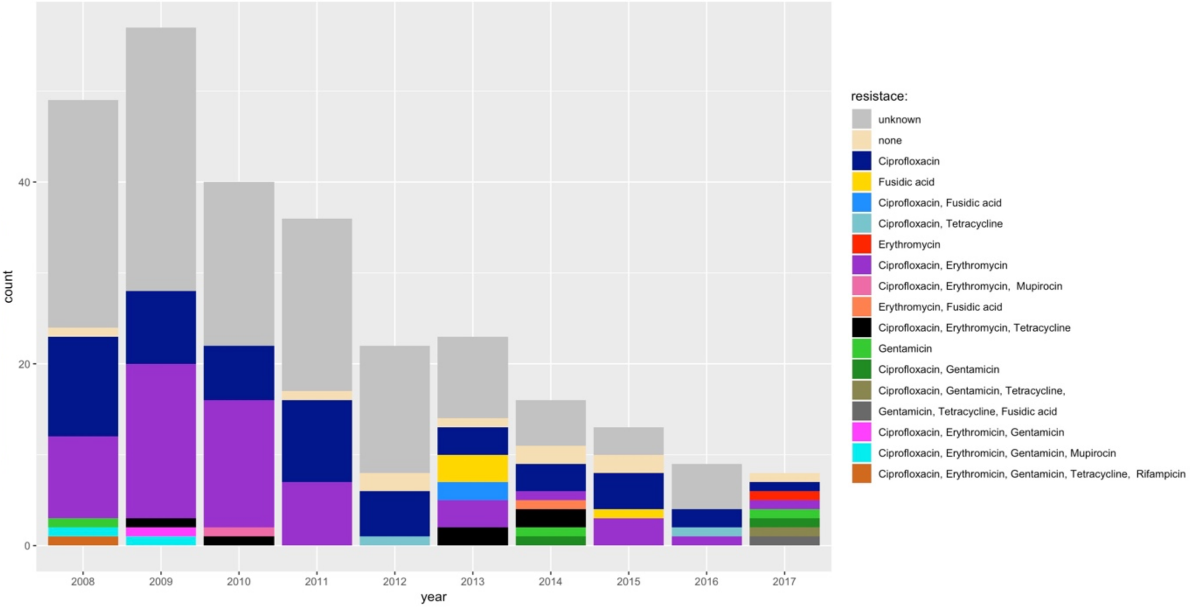

Supplementary Figure 7 Number of patients with a positive test for each year of the study. The data collection started in mid 2008 and ended in mid 2017. The colours correspond to the unique

resistance profiles assigned to each patient based on 7 antibiotics (Gentamycin, Erythromycin, Tetracycline, Fusidic acid, Ciprofloxacin, Rifampicin, Mupirocin.)

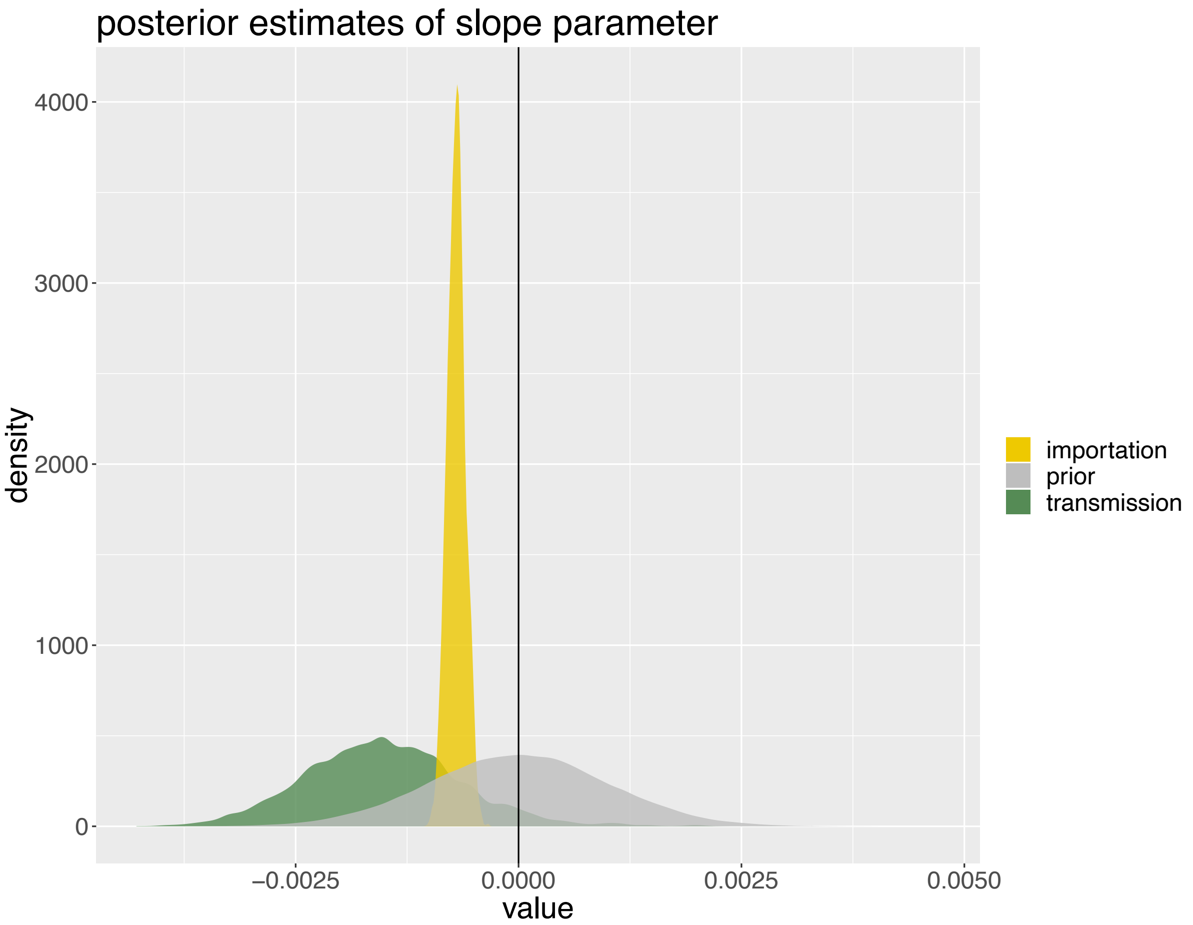

Supplementary Figure 8. Posterior estimates of the slope parameter of the time dependent transmission and importation. Negative values indicate a decrease over time. The data plotted are for the model using typing data.

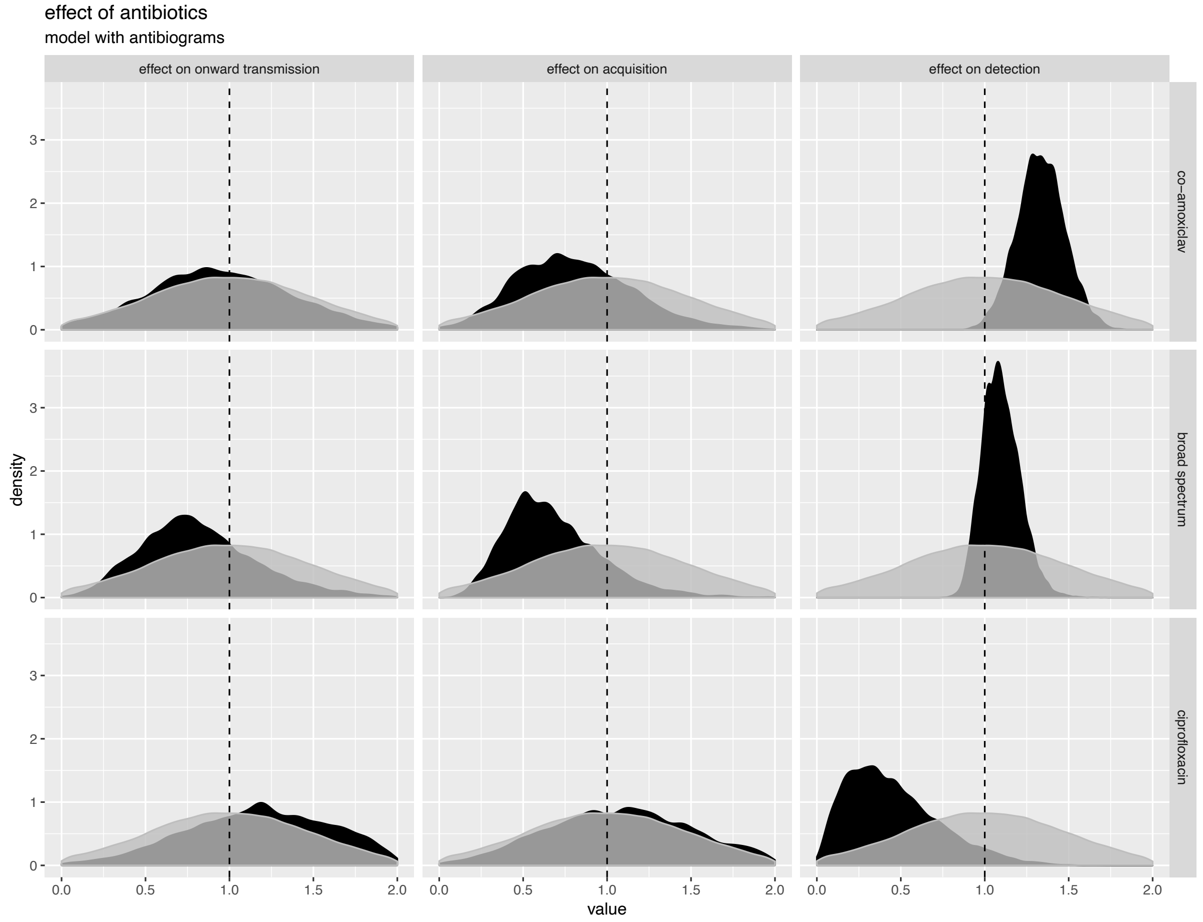

Supplementary Figure 9. Posterior estimates of the model with antibiograms (black) and prior distributions (grey) of the effects of antibiotics on acquisition, onward transmission and detection in an Oxford ICU, 2008-2017.

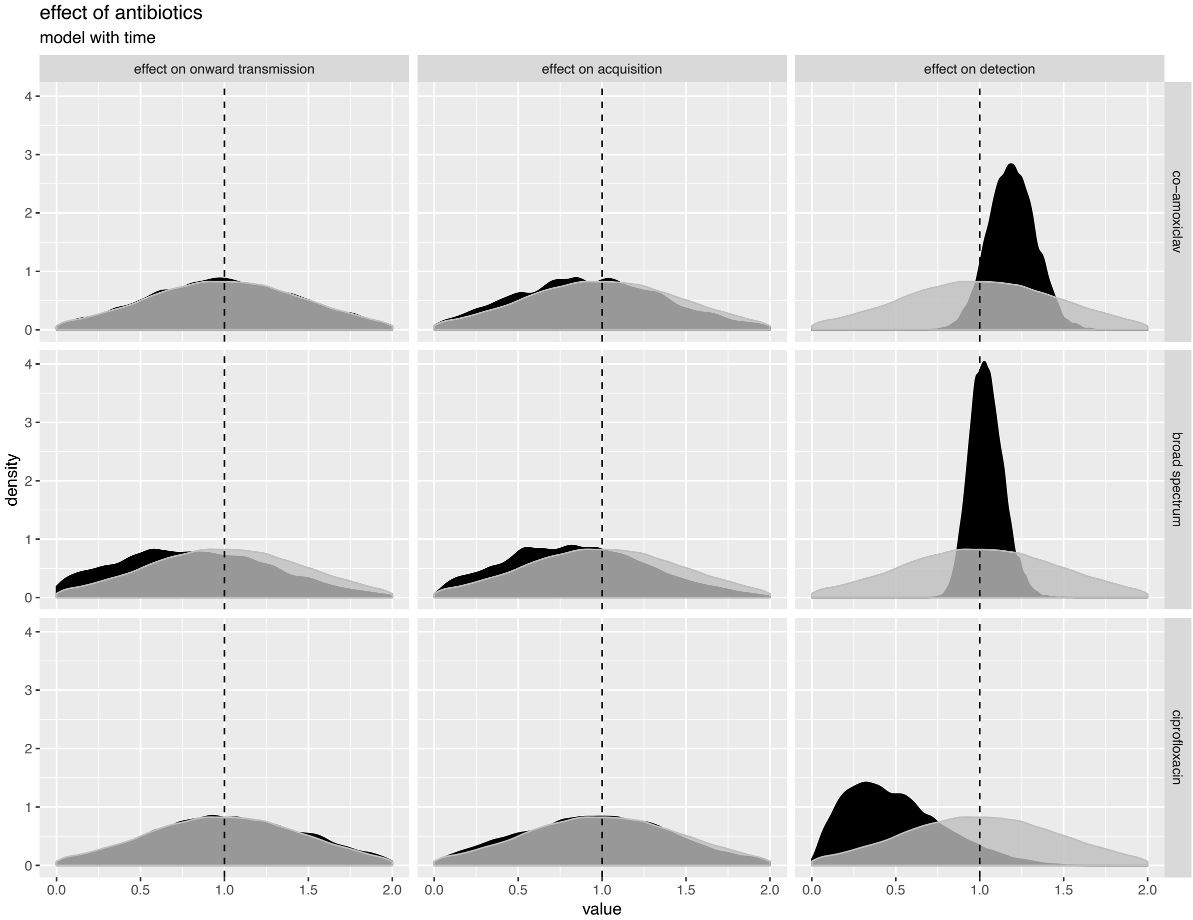

Supplementary Figure 10. Posterior estimates of the model with time (black) and prior distributions (grey) of the effects of antibiotics on acquisition, onward transmission and detection in an Oxford ICU, 2008-2017.

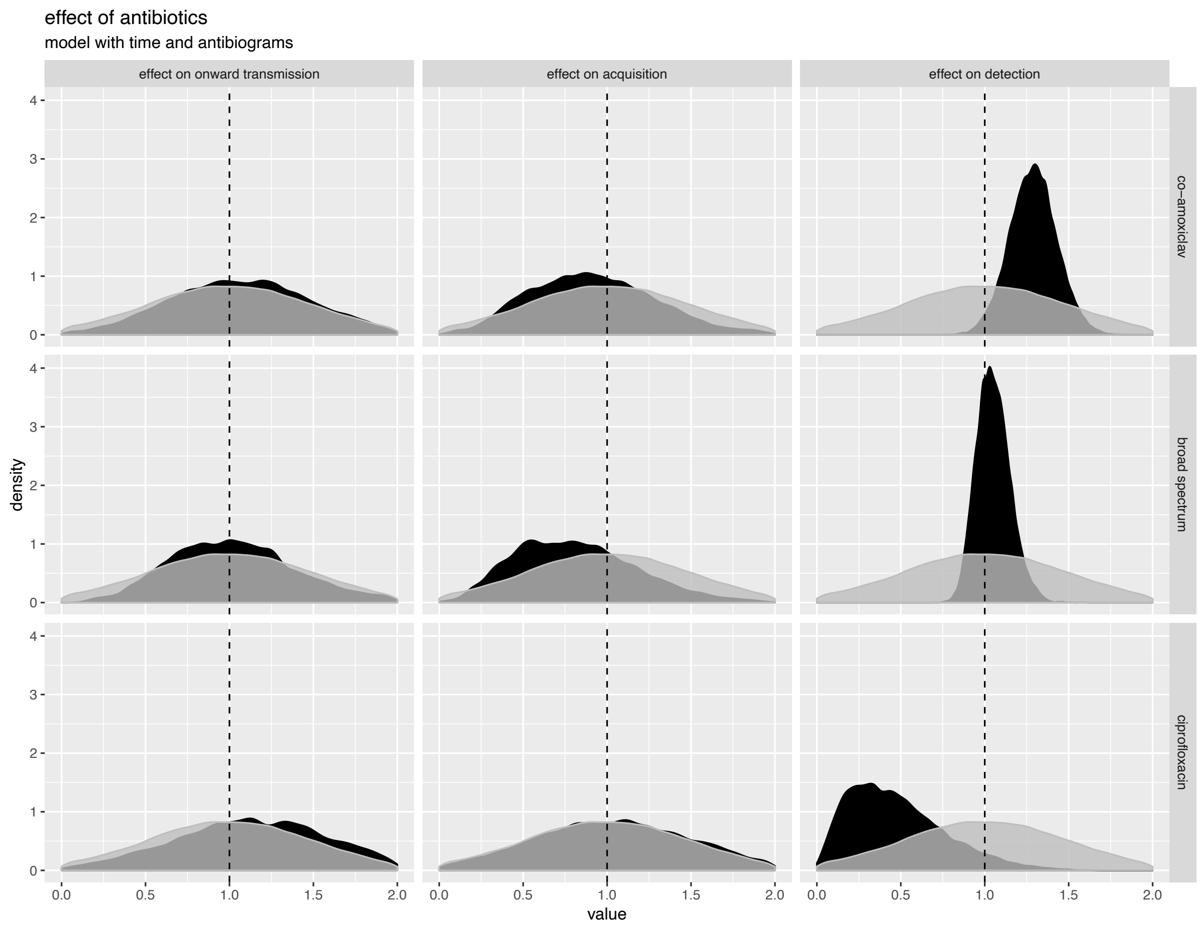

Supplementary Figure 11. Posterior estimates of the model with time and antibiograms (black) and prior distributions (grey) of the effects of antibiotics on acquisition, onward transmission and detection in an Oxford ICU, 2008-2017.

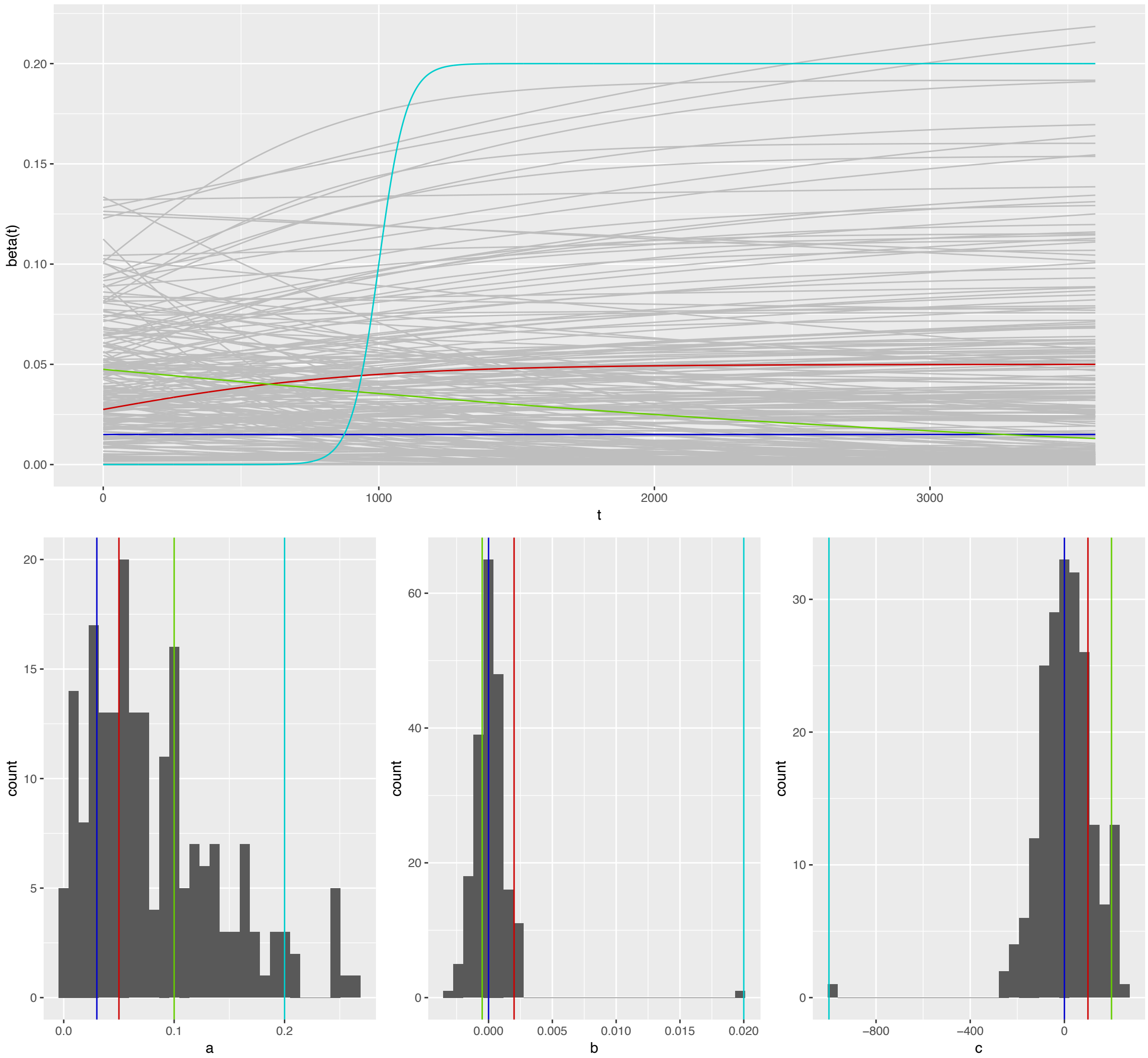

Supplementary Figure 12. Example of possible curves for the time dependent transmission and importation parameter. The bottom row shows values sampled from the priors of the shape parameters a (upper asymptote), b (slope) and c (horizontal shift). Each grey line in the top plot corresponds to one combination of the values sampled from the prior. The values indicated by the coloured vertical line were chosen to show examples of possible curves, such as no change (blue), linear decrease (green), increase and saturation (red) and sudden transition (light blue).

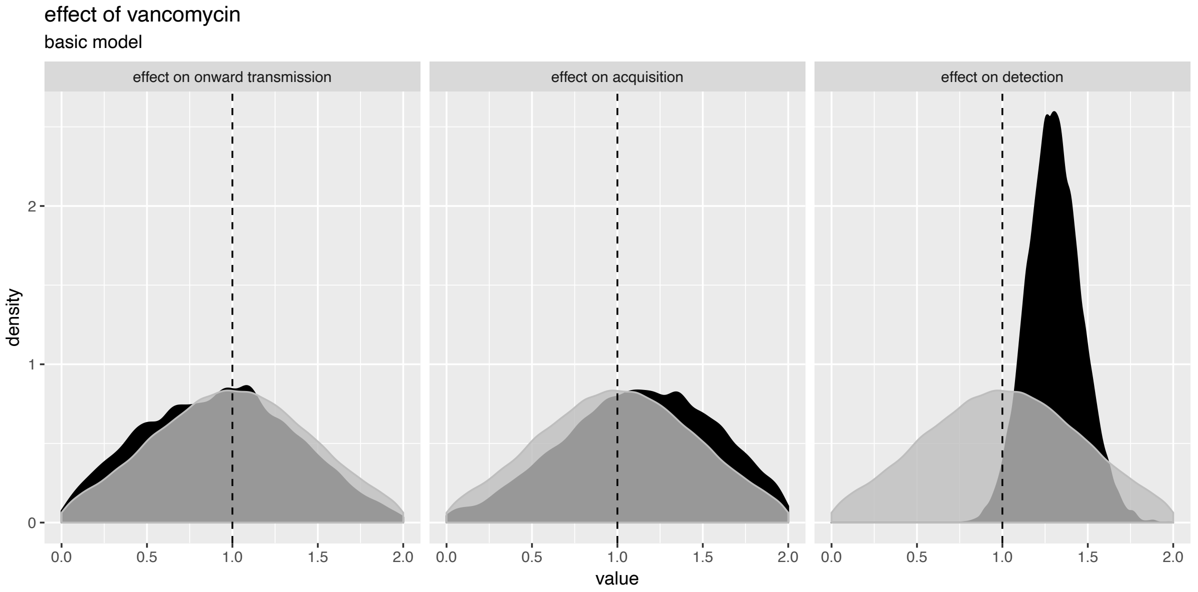

Supplementary Figure 13 Posterior estimates of the basic model (black) and prior distributions (grey) of the effects of vancomycin on acquisition, onward transmission and detection in an Oxford ICU, 2008-2017.

Supplementary Tables:

**number of patients**	7924
**age (median, [IQR])**	64 [47, 74]
**Charlson comorbidity score (median, [IQR])**	3 [0, 9]
**Sex (N, %)**	
male	4777 (60%)
female	3177 (40%)
**admission speciality group (N, %)**	
acute medicine	1712 (22%)
specialist medicine	1405 (18%)
general surgery	2475 (31%)
trauma and orthopaedics	1128 (14%)
other	1204 (15%)

Supplementary Table 1. Summary of characteristics of patients admitted to the combined medical and surgical adult ICU between June 2008 and November 2017.

	importation		acquisition		onward transmission	
	multivariate OR (95 % CI)	p-value	multivariate OR (95 % CI)	p-value	multivariate OR (95 % CI)	p-value
**age** (per 10 year increase)	1.11 (1.01, 1.22)	0.04	1.05 (0.85, 1.29)	0.68	1.20 (0.86, 1.69)	0.29
**Charlson comorbidity score** (per unit increase)	0.99 (0.97, 1.01)	0.47	0.99 (0.95, 1.04)	0.75	0.95 (0.88, 1.03)	0.21
**sex**						
female	1.00		1.00		1.00	
male	1.12 (0.82, 1.55)	0.47	1.47 (0.69, 3.12)	0.32	2.31 (0.69, 7.75)	0.18
**admission speciality group**						
acute medicine	1.00		1.00		1.00	
specialist medicine	1.04 (0.66, 1.64)	0.85	1.62 (0.56, 4.70)	0.37	0.99 (0.22, 4.44)	0.99
general surgery	0.80 (0.53, 1.22)	0.31	1.15 (0.42, 3.12)	0.78	0.96 (0.25, 3.76)	0.96
T&O	0.52 (0.28, 0.97)	0.04	0.74 (0.18, 3.00)	0.67	0.54 (0.05, 5.53)	0.60
other	0.70 (0.40, 1.20)	0.19	0.95 (0.27, 3.40)	0.94	1.06 (0.17, 6.81)	0.95

Supplementary Table 2. Relationship between importation, acquisition and onward transmission and patient factors. There was no evidence of non-linearity using multiple fractional polynomials.
